# Supplementary material for: Essential role of PLD2 in hypoxia-induced stemness and therapy resistance in ovarian tumors
Source: J Exp Clin Cancer Res. 2024 Feb 26;43:57. doi: 10.1186/s13046-024-02988-y (PMC10895852; doi:10.1186/s13046-024-02988-y)
Supplement: Supplementary file 1 — Supplementary Material 1 [file 13046_2024_2988_MOESM1_ESM.pdf]

Supplemental Information

Muñoz-Galván et al., "Essential role of PLD2 in hypoxia-induced stemness and therapy resistance in ovarian tumors"

Table S1. Reagents used in this work.

**Antibody**	**Dilution**	**Reference**
Anti-PLD2 (E1Y9G)	1:1000 (Wb)/1:250(IF)	Cell Signaling ##13904
Anti-HIF1A	1:500 (Wb)/ 1:200 (IF)	CaymanChem#10006421
Anti-SOX9	1:200	Abcam # ab185230
Anti-NOTCH1	1:250	SantaCruz #sc-6014-r
Anti-Sox-2 (E-4)	1:250	SantaCruz # sc-365823
Anti-SOX17 [EPR20684]	1:250	Abcam #ab224637
mAb anti-α-tubulin	1:5000	Sigma 9026
peroxidase-labeled rabbit anti-mouse	1:10000	Amersham
peroxidase-labeled goat anti-rabbit	1:10000	Abcam #6721
**Probe**		**Reference**
PLD2		ThermoFisher#Hs00160163
LDHA		ThermoFisher#Hs01378790
VEGFA		ThermoFisher#Hs00900055
SOX2		ThermoFisher#Hs01053049
NANOG		ThermoFisher#Hs04260366
CD44		ThermoFisher#Hs01075861
EPCAM		ThermoFisher#Hs00901885
SOX9		ThermoFisher#Hs01001343
NOTCH1		ThermoFisher#Hs01062014
SNAI1		ThermoFisher#Hs00195591
VIM		ThermoFisher#Hs00958111
CDH1		ThermoFisher#Hs01023894
CDH2		ThermoFisher#Hs00983056
ACTB		ThermoFisher#Hs001060665

Table S2: Patient Cohort characteristics

	Sensitive<br>N=10 (40%)	Resistant<br>N=15(60%)
**Age (years)**<br>• Mean (Rank)	 62,0 (34-70)	 51,0 (40-67)
**ECOG**<br>• 0<br>• 1<br>• 2	 7 (77,8%)<br>1 (11,1%)<br>1 (11,1%)	 5 (38,5%)<br>6 (46,2%)<br>2 (15,4%)
**Stage (FIGO 2014)**<br>• IA<br>• IC<br>• IIB<br>• IIIB<br>• IIIC<br>• IVA<br>• IVB	 1 (11,1%)<br>1 (11,1%)<br>1 (11,1%)<br>1 (11,1%)<br>4 (44,4%)<br>1 (11,1%)<br>0	 1 (7,7%)<br>1 (7,7%)<br>0<br>1 (7,7%)<br>8 (61,5%)<br>0<br>2 (15,4%)
**Ca 125 (U/ml)**<br>• **Diagnosis:** Median (Rank)<br>• **After treatment:** Median (Rank)	 194 (31,6-21957)<br>11 (6,5-1400)	 332 (38-3892)<br>76,1 (15,5-1862)
**Adjuvant Chemotherapy**<br>• No<br>• Yes	 6 (66,7%)<br>3 (33,3%)	 7 (53,8%)<br>6 (46,2%)
**Treatment**<br>• Carbo + Paclitaxel<br>• Carbo + Paclitaxel + beva<br>• Carbo monotherapy	 6 (68%)<br>3 (32%)<br>0	 12 (92%)<br>0<br>1 (8%)
**Surgery**<br>• R0<br>• R1<br>• Biopsies<br>• No (incluyen pacientes con cirugía primaria)	 1 (11,1%)<br>2 (22,2%)<br>0<br>6 (66,7%)	 2 (15,4%)<br>2 (15,4%)<br>2 (15,4%)<br>7 (53,8%)
**Better response to QT adyuvant or 1st líne**		

• RC<br>• RP<br>• EE<br>• PE	6 (66,7%)<br>3 (33,3%)<br>0<br>0	4 (30,8%)<br>2 (15,4%)<br>2 (15,4%)<br>5 (38,5%)
**Treatment after 1st line**<br>• Bevacizumab<br>• Others<br>• No	 3 (33,3%)<br>0<br>6 (66,7%)	 0<br>0<br>13 (100%)
**Progression disease after treatment**<br>• yes<br>• No	 6 (66,7%)<br>3 (33,3%)	 13 (100%)<br>0
**Platinum free interval (moths)**<br>• Mean (Rank)	 19 (7-33)	 1 (0-5)
**More than 2 lines of treatment**<br>• yes<br>• No	 4 (44,4%)<br>5 (55,6%)	 4 (30,8%)<br>9 (69,12%)
**Status patient in last visit**<br>• Live without disease<br>• Live with disease<br>• Death (all due to disease progression)	 2 (22,2%)<br>4 (44,4%)<br>3 (33,3%)	 0<br>2 (15,4%)<br>11 (84,6%)
**Location primary tumor**<br>• Right ovary<br>• Left ovary<br>• Bilateral<br>• Peritoneal	 2 (22,2%)<br>2 (22,2%)<br>5 (55,6%)<br>0	 2 (15,4%)<br>4 (30,8%)<br>5 (38,5%)<br>2 (15,4%)
**Differentiation**<br>• Moderately<br>• Poor<br>• nd	 1 (11,1%)<br>7 (77,8%)<br>1 (11,1%)	 1 (7,7%)<br>11 (84,6%)<br>1 (7,7%)
**Histology and Subtype of OC**<br>• HGSOC<br>• Clear cell carcinoma<br>• Endometroid	 7 (77,8%)<br>2 (22,2%)<br>0	 8 (61,5%)<br>3 (23,1%)<br>2 (15,4%)
**Lymphovascular infiltration**<br>• No<br>• Yes<br>• nd	 1 (11,1%)<br>2 (22,2%)	 1 (7,7%)<br>1 (7,7%)

	6 (66,7%)	11 (84,6%)
**BRCA Mutation**<br>• No<br>• Yes<br>• nd	 4 (44,4%)<br>2 (22,2%)<br>3 (33,3%)	 6 (46,2%)<br>0<br>7 (53,8%)

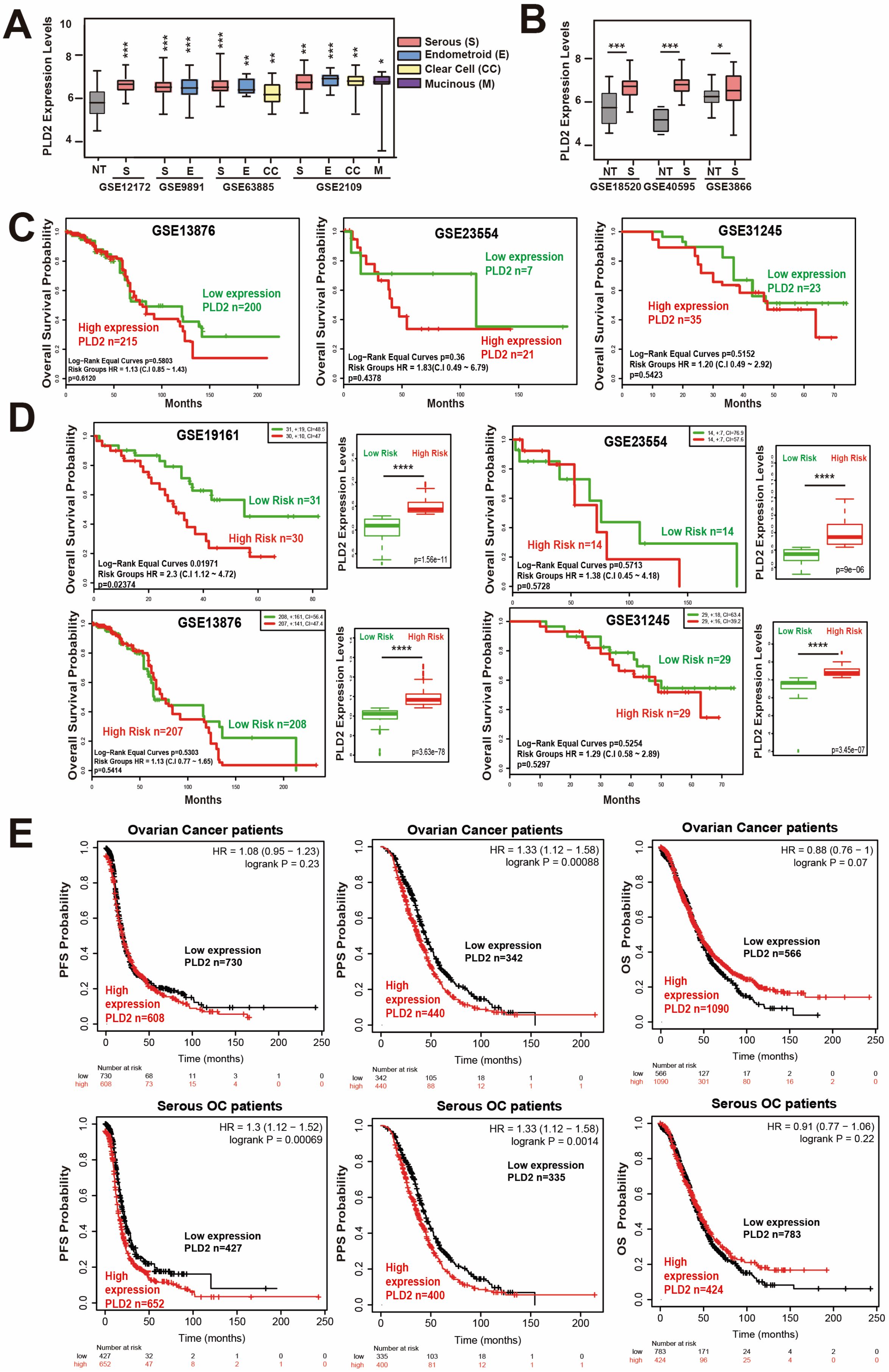
A
PLD2 Expression Levels
Serous (S)
Endometroid (E)
Clear Cell (CC)
Mucinous (M)
NT S S E S E CC S E CC M
GSE12172 GSE9891 GSE63885 GSE2109
B
PLD2 Expression Levels
NT S NT S NT S
GSE18520 GSE40595 GSE3866
C
Overall Survival Probability
GSE13876
Low expression PLD2 n=200
High expression PLD2 n=215
Log-Rank Equal Curves p=0.5803
Risk Groups HR = 1.13 (C.I 0.85 ~ 1.43)
p=0.6120
Months
GSE23554
Low expression PLD2 n=7
High expression PLD2 n=21
Log-Rank Equal Curves p=0.36
Risk Groups HR = 1.83(C.I 0.49 ~ 6.79)
p=0.4378
Months
GSE31245
Low expression PLD2 n=23
High expression PLD2 n=35
Log-Rank Equal Curves p=0.5152
Risk Groups HR = 1.20 (C.I 0.49 ~ 2.92)
p=0.5423
Months
D
Overall Survival Probability
GSE19161
Low Risk n=31
High Risk n=30
Log-Rank Equal Curves 0.01971
Risk Groups HR = 2.3 (C.I 1.12 ~ 4.72)
p=0.02374
PLD2 Expression Levels
Low Risk High Risk
p=1.56e-11
GSE23554
High Risk n=14
Low Risk n=14
Log-Rank Equal Curves p=0.5713
Risk Groups HR = 1.38 (C.I 0.45 ~ 4.18)
p=0.5728
p=9e-06
GSE13876
High Risk n=207
Low Risk n=208
Log-Rank Equal Curves p=0.5303
Risk Groups HR = 1.13 (C.I 0.77 ~ 1.65)
p=0.5414
Months
p=3.63e-78
GSE31245
Low Risk n=29
High Risk n=29
Log-Rank Equal Curves p=0.5254
Risk Groups HR = 1.29 (C.I 0.58 ~ 2.89)
p=0.5297
Months
p=3.45e-07
E
Ovarian Cancer patients
PFS Probability
HR = 1.08 (0.95 − 1.23)
logrank P = 0.23
Low expression PLD2 n=730
High expression PLD2 n=608
Time (months)
Number at risk
low 730 68 11 3 1 0
high 608 73 15 4 0 0
Ovarian Cancer patients
PPS Probability
HR = 1.33 (1.12 − 1.58)
logrank P = 0.00088
Low expression PLD2 n=342
High expression PLD2 n=440
Time (months)
Number at risk
low 342 105 18 1 0
high 440 88 12 1 1
Ovarian Cancer patients
OS Probability
HR = 0.88 (0.76 − 1)
logrank P = 0.07
Low expression PLD2 n=566
High expression PLD2 n=1090
Time (months)
Number at risk
low 566 127 17 2 0 0
high 1090 301 80 16 2 0
Serous OC patients
PFS Probability
HR = 1.3 (1.12 − 1.52)
logrank P = 0.00069
Low expression PLD2 n=427
High expression PLD2 n=652
Time (months)
Number at risk
low 427 32 2 1 0 0
high 652 47 8 2 1 0
Serous OC patients
PPS Probability
HR = 1.33 (1.12 − 1.58)
logrank P = 0.0014
Low expression PLD2 n=335
High expression PLD2 n=400
Time (months)
Number at risk
low 335 103 18 1 0
high 400 81 12 1 1
Serous OC patients
OS Probability
HR = 0.91 (0.77 − 1.06)
logrank P = 0.22
Low expression PLD2 n=783
High expression PLD2 n=424
Time (months)
Number at risk
low 783 171 24 4 2 0
high 424 96 25 4 0 0

Figure S1. *PLD2* expression in OC patients and patient survival in OC public databases. (**A**) *PLD2* expression in the OC patient databases GSE12172, GSE9891, GSE63885 and GSE2109 indicating OC subtype. (**B**) *PLD2* expression in the OC patient databases GSE18520, GSE40595, and GSE38666, indicating OC subtype. (**C**) Kaplan-Meier plots showing overall survival (OS) of patients with high (red) or low (green) *PLD2* expression levels in three OC databases with survival data: GSE13876 (advanced HGSOC); GSE23554 (advanced serous epithelial OC); and GSE31245 (92% serous, 2% endometroid, 6% clear cell). Data were analyzed with the log-rank test, and the associated P-values are shown in the graphs. (**D**) Kaplan-Meier plots showing overall survival (OS) of patients with high (red) or low (green) risk. *PLD2* expression for each group is shown on the right of each graph. (**E**) Kaplan-Meier plots generated with Kaplan-Meier Plotter showing PFS (left column), PPS (middle column) and OS (right column) by splitting patients according to *PLD2* expression in all OC patients (top row) or only HGSOC patients (bottom row). Expression levels are shown as $\log_2$ transformed values from the R2 database. Data were analyzed using Student's *t*-test. *, P < 0.05; **, P < 0.01; ***, P < 0.001.

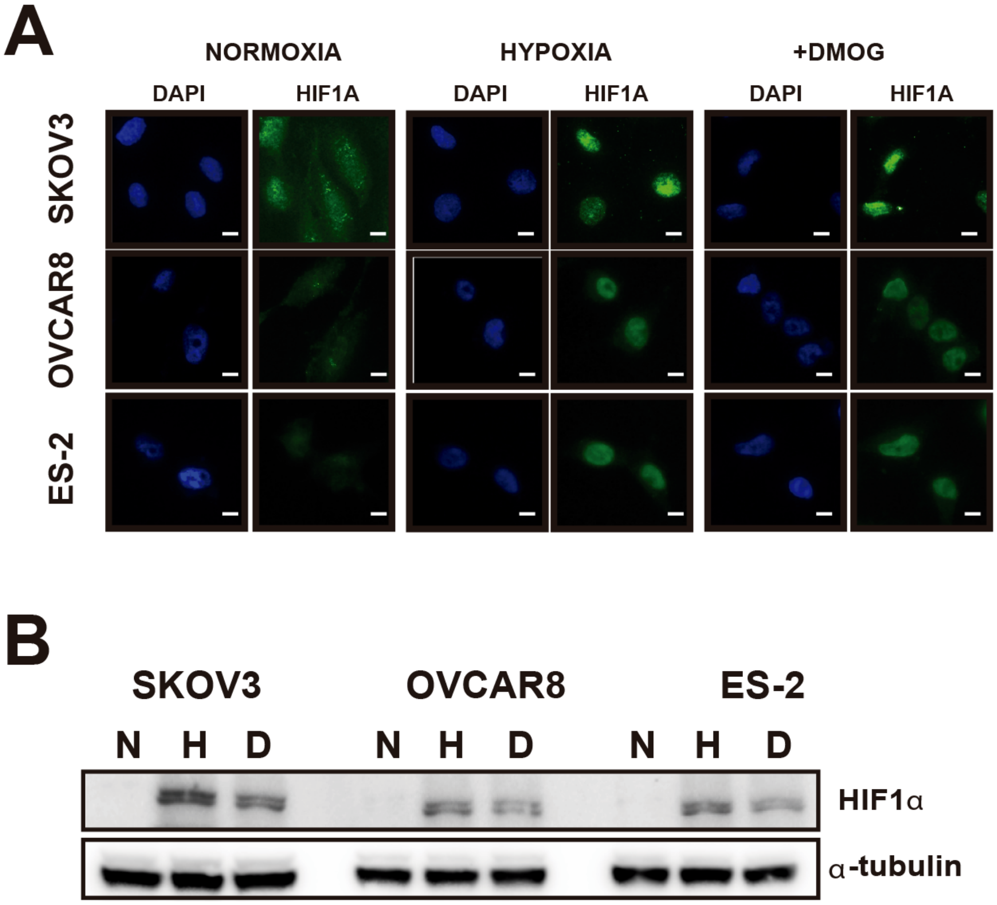

Figure S2. HIF1α levels in OC cell lines in response to hypoxia. **(A)** Representative images of HIF-1α protein levels by immunofluorescence in SKOV3, OVCAR8 and ES-2 cells under normoxia and hypoxia or in the presence of the HIF-hydroxylase inhibitor DMOG. **(B)** Western blot showing HIF-1α and alpha-tubulin protein levels in SKOV3, OVCAR8 and ES-2 cells under normoxia and hypoxia or in the presence of the HIF-hydroxylase inhibitor DMOG. A minimum of three independent experiments were performed.

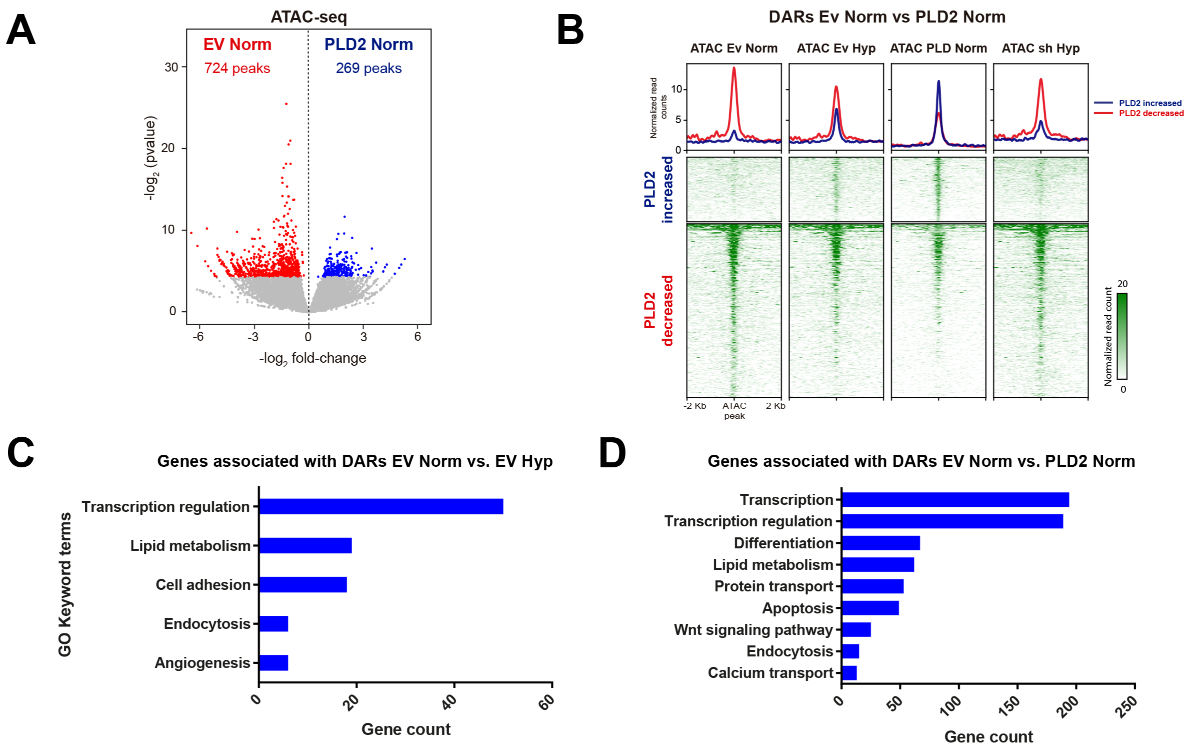

Figure S3. Differential analyses of chromatin accessibility in OC cells. **(A)** Volcano plot showing differential analyses of chromatin accessibility between SKOV3 cells carrying Ev and plasmid overexpressing *PLD2* in normoxia. **(B)** Heatmaps and average profiles plotting normalized ATAC-seq signal in SKOV3 cells carrying Ev and overexpressing *PLD2* for the differentially accessible regions (DARs) in (A). **(C)** Gene Ontology term enrichment analyses of biological processes for the genes associated with DARs in SKOV3 cells carrying EV in normoxia versus hypoxia. **(D)** Gene Ontology term enrichment analyses of biological processes for the genes associated with DARs in SKOV3 cells carrying Ev versus overexpressing *PLD2* in normoxia.

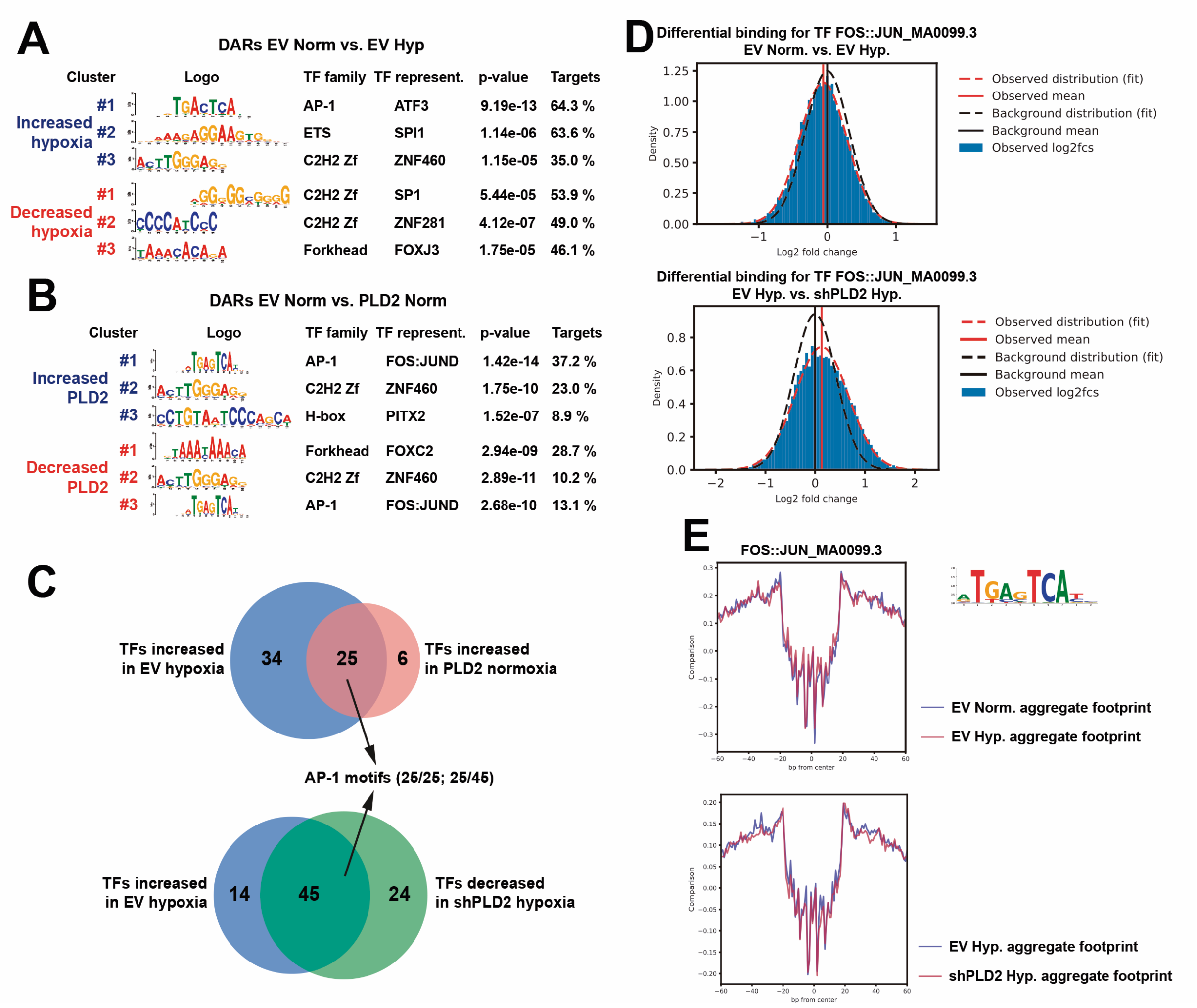

Figure S4. Motif and footprint analyses of transcription factor binding in OC cells. **(A-B)** Motif enrichment analyses of the increased and decreased ATAC peaks in OC cells carrying Ev in hypoxia vs. normoxia (A) and +/- *PLD2* expression in normoxia (B). The three motifs with the lowest p values are shown in each case. **(C)** Venn diagrams plotting the overlap between TFs with increased binding in hypoxia and expressing *PLD2* in normoxia (top) or the overlap between TFs with increased binding in hypoxia and expressing *shPLD2* in hypoxia (bottom). **(D)** Distribution of fold changes in the ATAC peaks containing the motifs FOS::JUN in Ev normoxia vs. Ev hypoxia (top) or in Ev hypoxia vs. *shPLD2* hypoxia**.** **(E)** Aggregate footprint signal of the peaks containing the motifs in (D).

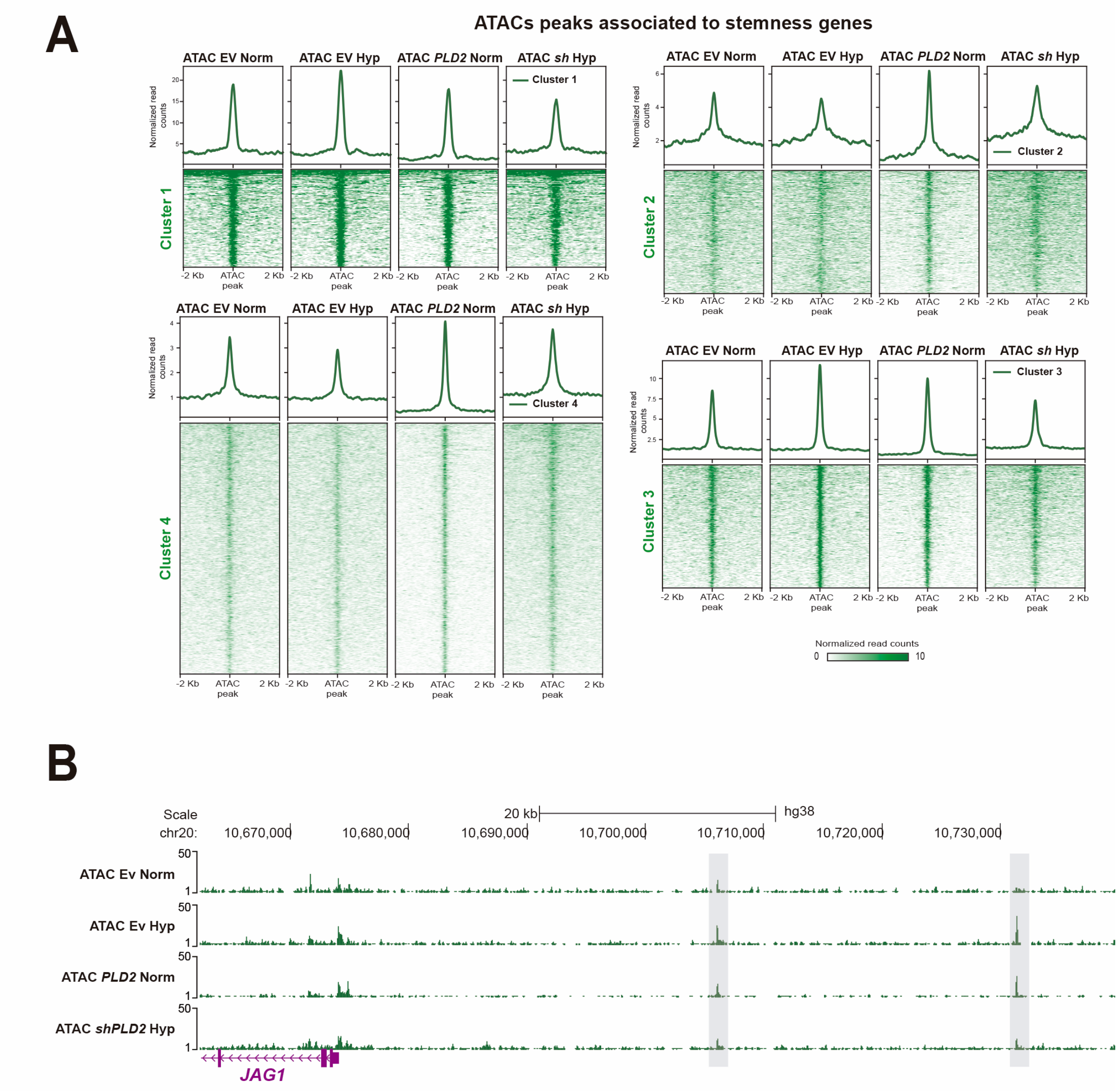

Figure S5. Clustering of differentially accessible regions in OC cells. **(A)** Heatmaps plotting normalized ATAC-seq signal at peaks associated with stemness genes in SKOV3 cells carrying Ev or plasmid expressing *PLD2* in normoxia and carrying Ev or plasmid expressing *shPLD2* in hypoxia, for the differentially accessible regions (DARs) clustered using *k*-means method in 4 clusters. **(B)** Tracks with ATAC-seq in SKOV3 cells carrying Ev or expressing *PLD2* in normoxia and carrying Ev or *shPLD2* in hypoxia, at the *JAG1* locus.

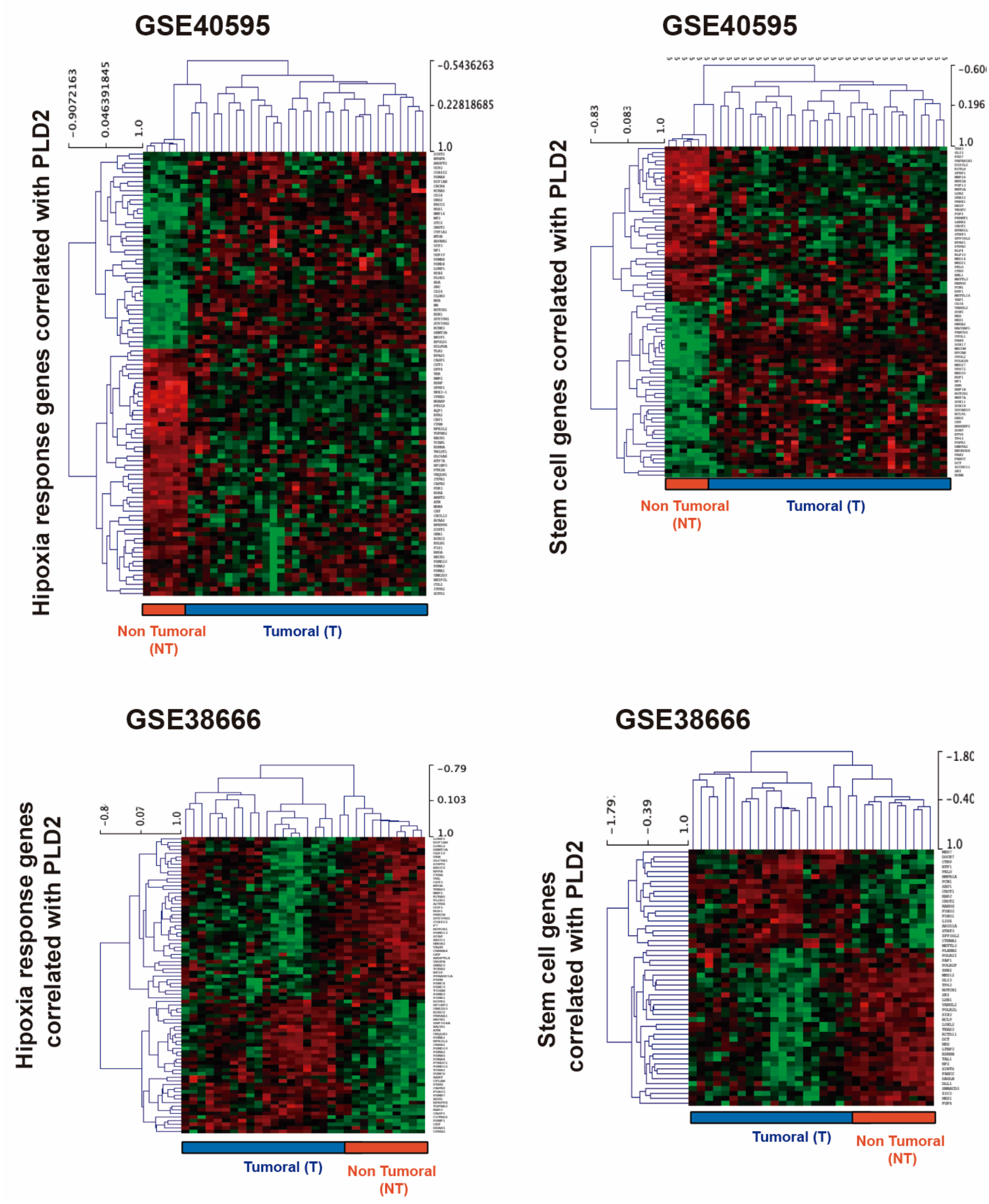

Figure S6. Expression of stemness and hypoxia genes correlated with *PLD2* in OC patients. Heatmaps showing the expression z-scores of stemness-associated genes or hypoxia-response genes whose expression correlated with *PLD2* in GSE40595 and GSE38666 OC patient databases.

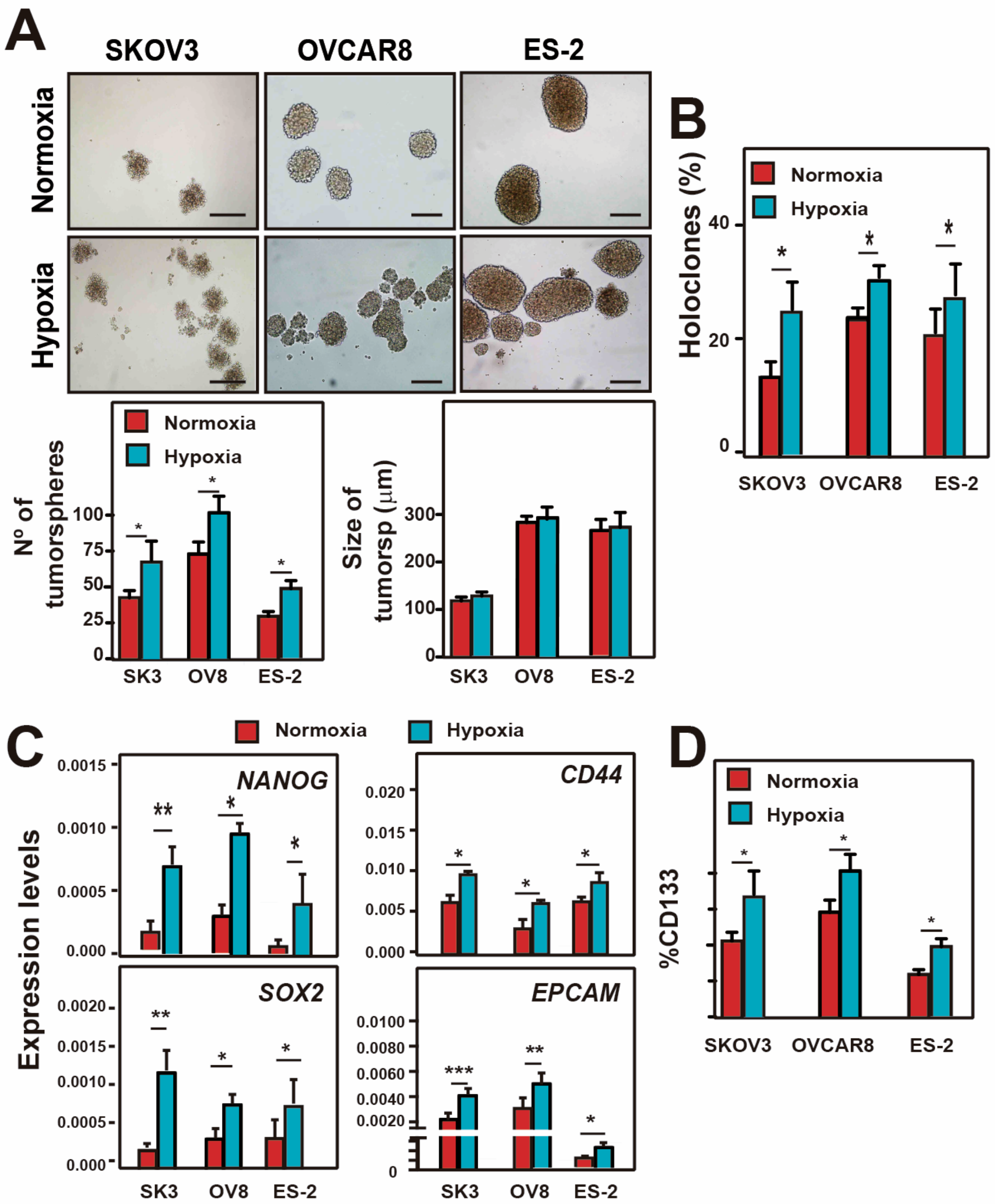

Figure S7. Hypoxia induces CSCs in ovarian cancer cells. **(A)** Top, Representative images of tumorspheres formed by SKOV3, OVCAR8 and ES-2 cells in normoxia or hypoxia. Bottom, quantification of the number and size of tumorspheres. Scale bars: 250 μm. **(B)** Percentage of holoclones formed by SKOV3, OVCAR8 and ES-2 cells in normoxia or hypoxia. At least 200 individual clones were analyzed. (**C**) Analysis of the expression of *NANOG*, *SOX2*, *CD44* and *EPCAM* stemness-associated genes by RT-qPCR in SKOV3, OVCAR8 and ES-2 cells in normoxia or hypoxia. The mRNA expression was calculated as $2^{-\Delta Ct}$ relative to the *ACTB* gene. **(D)** Percentage of CD133 positive cells measured by FACS in SKOV3, OVCAR8 and ES-2 cells in normoxia and hypoxia The average and SD of three independent experiments are shown in all cases. A minimum of three independent experiments were performed and the data were compared using Student's t tests. Asterisks indicate statistical significance with respect to normoxia. $^*p < 0.05$; $^{**}p < 0.01$; $^{***}p < 0.001$.

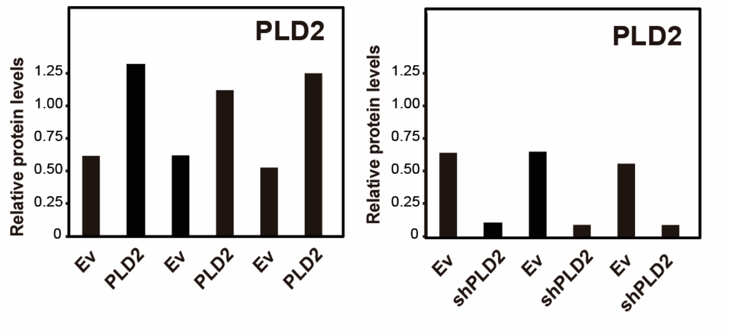

Figure S8. Relative protein quantification of PLD2 normalized to alpha-tubulin from the western blots in Figure 4B.

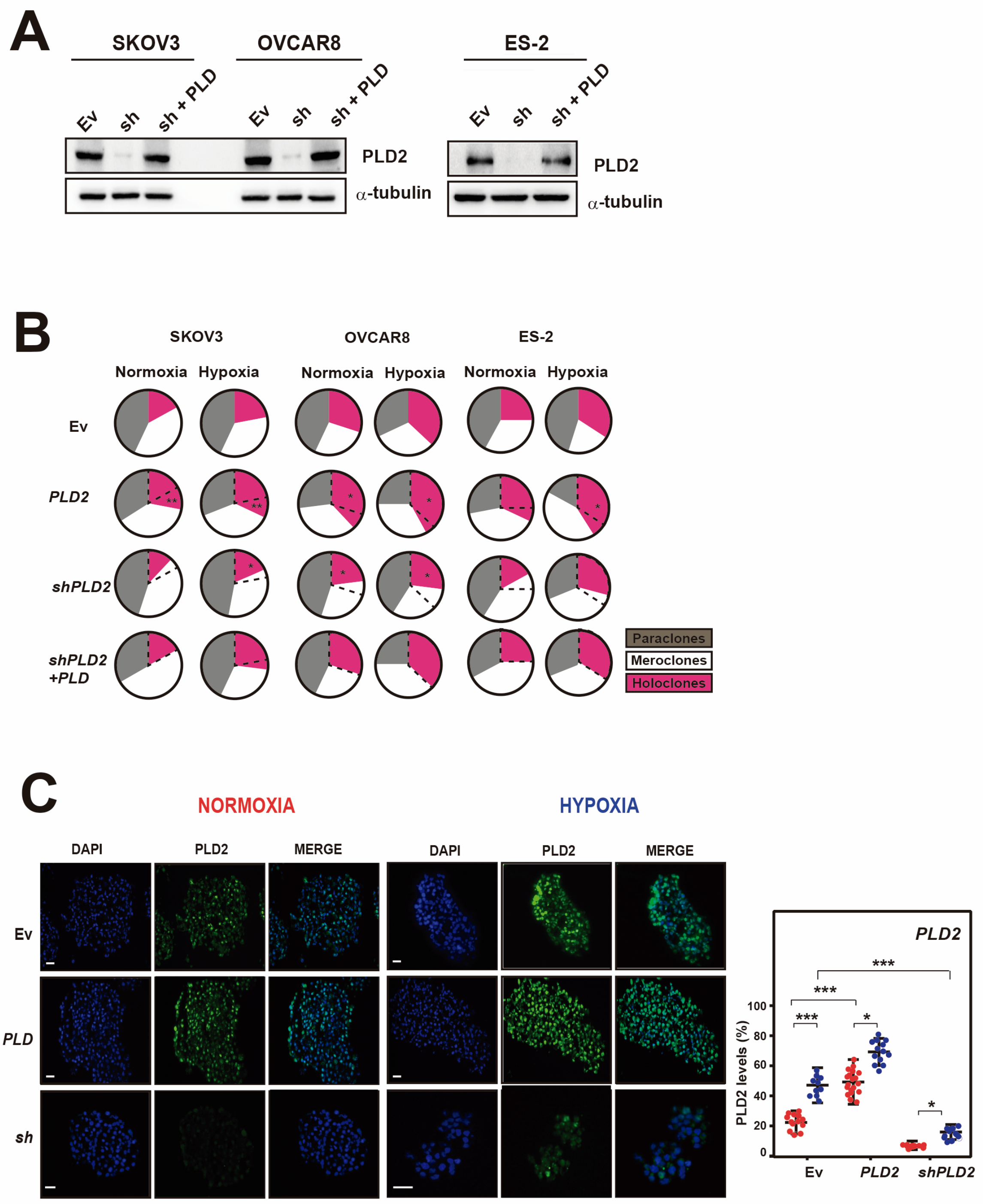

Figure S9. PLD2 expression and clone analyses. (**A**) Western blot showing PLD2 and alpha-tubulin protein levels in SKOV3, OVCAR8 and ES-2 OC cells carrying Ev, a plasmid expressing *shPLD2* or plasmids expressing *shPLD2* and *PLD2*. (**B**) Percentage of paraclones, meroclones and holoclones formed by SKOV3, OVCAR8 and ES-2 cells carrying Ev or plasmids expressing *PLD2*, *shPLD2* or both in hypoxia or normoxia. At least 200 individual clones were analyzed. The average of three independent experiments is shown. A dotted line represents the percentage of holoclones in Ev carrying cells as a reference. Data were compared using Student's t tests. Asterisks indicate statistical significance with respect to Ev carrying cells. *$p < 0.05$. (**C**) Left, determination of PLD2 protein levels by immunofluorescence in tumorspheres formed by OC cells carrying Ev and expressing *PLD2* or *shPLD2*. Right, quantification of the percentage of cells with PLD2 expression in tumorspheres. Scale bars: 100 µm.

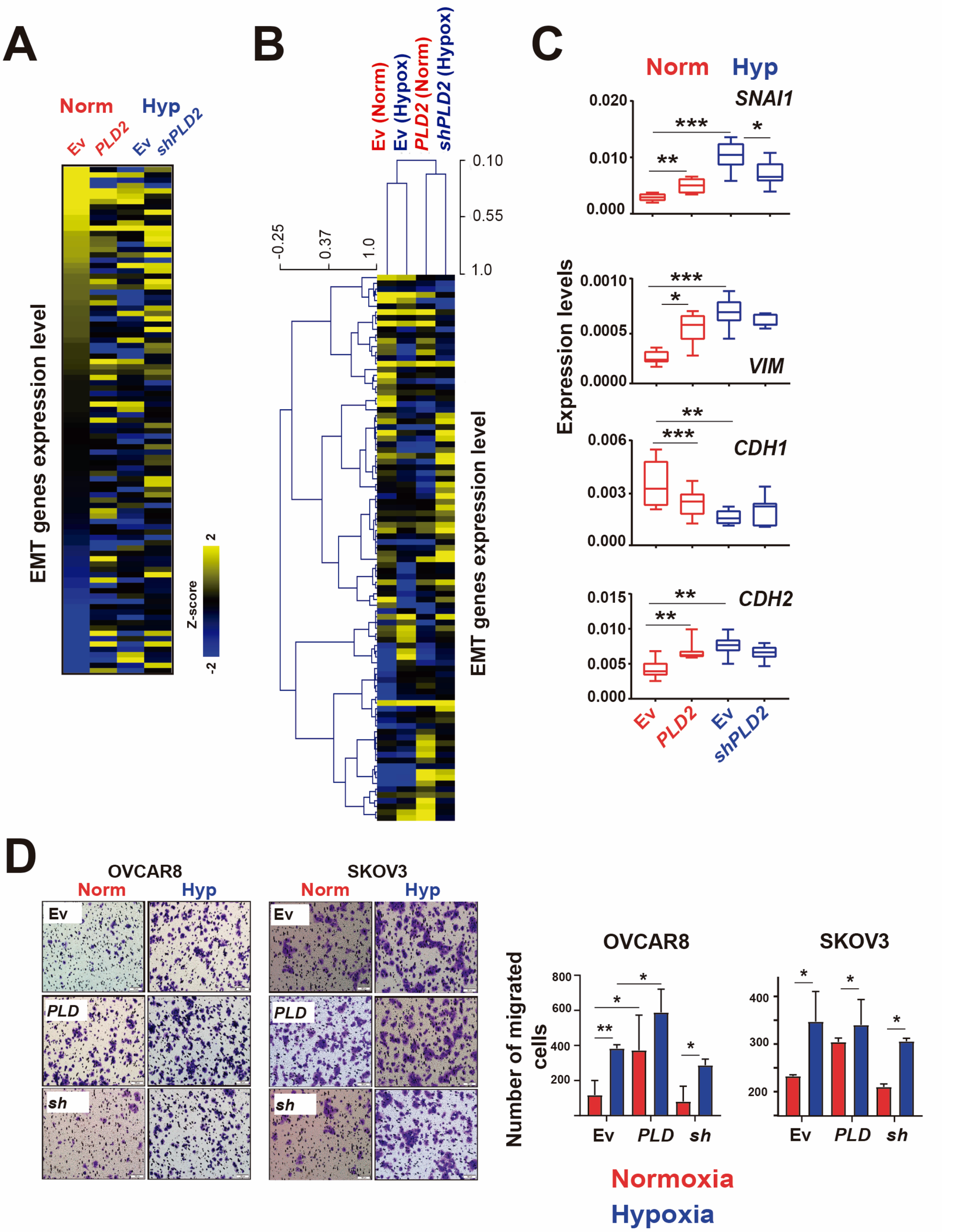

Figure S10. Analyses of the EMT in OC cells in response to hypoxia and/or *PLD2* expression. **(A)** Heatmaps showing the expression z-scores of epithelial-to-mesenchymal transition-associated genes obtained from TaqMan Arrays. Genes are sorted according to decreasing z-scores in the Ev-carrying cells under normoxia. **(B)** Heatmaps showing the z-scores of EMT genes expression levels in SKOV3 cells carrying EV or plasmid overexpressing *PLD2* under normoxia conditions and carrying EV or plasmid expressing *shPLD2* under hypoxia condition. Hierarchical clustering of the samples is shown. **(C)** Expression levels of *SNAI1, VIM, CDH1* and *CDH2* EMT-associated genes in cells carrying Ev or plasmids expressing *PLD2* under normoxia or *shPLD2* under hypoxia conditions. **(D)** Left, representative images of the Boyden chamber migration assays in SKOV3 and OVCAR8 cells carrying Ev or plasmids expressing *PLD2* or *shPLD2* under normoxic or hypoxic conditions. Right, quantification of the Boyden chamber migration assays. A minimum of three independent experiments were performed and the data were analyzed using Student's *t*-test. *, $P < 0.05$; **, $P < 0.01$; ***, $P < 0.001$.
