# Supplementary material for: Essential role of PLD2 in hypoxia-induced stemness and therapy resistance in ovarian tumors
Source: J Exp Clin Cancer Res. 2024 Feb 26;43:57. doi: 10.1186/s13046-024-02988-y (PMC10895852; doi:10.1186/s13046-024-02988-y)
Supplement: Supplementary file 2 — Supplementary Material 2 [file 13046_2024_2988_MOESM2_ESM.docx]

**Point by point response to Reviewer reports.**

**Reviewer #2:**

**Comment:** Thank you for the revised manuscript; the revisions improved the clarity of the report. I have a few comments regarding edits:

-Abstract, line 26 (page 2): "Results Here, we show that hypoxia induces" appears transposed into the sentence "Such chemoresistance relies on the presence of cancer stem cells..." Please remove this sentence fragment and insert before line 31: "CSC formation and chemoresistance in ovarian cancer through transcriptional activation..."

**Response:** Thank you for bringing this to our attention. We have fixed the transposed sentence.

**Comment:** Results, lines 287-280 (page 11): "For this, we analysed PLD2 mRNA expression levels in 7 OC and one ovarian tissue databases using the R2 platform." This sentence is duplicated in the first half of the next sentence; this first sentence can be removed.

**Response:** We have removed the duplicated sentence. Thank you.

**Comment:** Figure 7A: the legend refers to scale bars, but I do not see any in Figure 7A. Please include scale bars in Figure 7A.

**Response:** We have added the scale bars to Figure 7A. Thank you.
